# Respiratory Syncytial Virus‐Associated Hospitalizations in Children: A 10‐Year Population‐Based Analysis in Finland, 2008–2018

**DOI:** 10.1111/irv.13268

**Published:** 2024-03-13

**Authors:** Erika Uusitupa, Matti Waris, Tytti Vuorinen, Terho Heikkinen

**Affiliations:** ^1^ Department of Pediatrics University of Turku and Turku University Hospital Turku Finland; ^2^ Department of Clinical Microbiology Turku University Hospital Turku Finland; ^3^ Institute of Biomedicine University of Turku Turku Finland

**Keywords:** children, hospitalization, infants, monoclonal antibodies, respiratory syncytial virus, vaccines

## Abstract

**Background:**

The risk of respiratory syncytial virus (RSV) hospitalization is highest during the first months of life, but few studies have assessed the population‐based rates of hospitalization in monthly age groups of infants.

**Methods:**

We determined the average population‐based rates of hospitalization with virologically confirmed RSV infections in children ≤15 years of age admitted during the 10‐year period of 2008–2018. Testing for RSV was routine in all children hospitalized with respiratory infections, and all RSV‐positive children admitted at any time during the study period were included in the analyses.

**Results:**

The annual population‐based rate of RSV hospitalization was highest in infants 1 month of age (52.0 per 1000 children; 95% CI, 45.2–59.7), followed by infants <1 month of age (34.8 per 1000; 95% CI, 29.2–41.1) and those 2 months of age (32.2 per 1000; 95% CI, 26.9–38.4). In cumulative age groups, the rate of hospitalization was 39.7 per 1000 (95% CI, 36.2–43.4) among infants <3 months of age, 26.8 per 1000 (95% CI, 24.8–29.0) in infants aged <6 months, and 15.8 per 1000 (95% CI, 14.7–17.0) in those <12 months of age.

**Conclusion:**

In monthly age groups of infants, the incidence rates of virologically confirmed RSV hospitalization in all infants up to 3 months of age were substantially higher than those reported in earlier studies. These data may be important for improving the estimates of the cost‐effectiveness of various interventions to reduce the burden of RSV in young infants.

## 1 Introduction

Respiratory syncytial virus (RSV) places a substantial burden of illness on children worldwide [1, 2, 3, 4, 5]. Globally, RSV‐associated morbidity and mortality are highest among children living in low‐ and middle‐income countries, but the impact of RSV is substantial also in high‐income countries in which approximately 2% of children are hospitalized with RSV during the first year of life [4, 5, 6, 7, 8]. Among infants, the hospitalization rates are uniformly highest in those <3 months of age, peaking at 1 month of age [9, 10, 11, 12, 13, 14, 15, 16, 17].

In recent years, crucial discoveries in the molecular structure of RSV have led to the development of an increasing number of potential interventions against RSV [18, 19, 20]. Several types of candidate vaccines and monoclonal antibodies are currently in different phases of development [21]. Nirsevimab, an extended half‐life monoclonal antibody, and a bivalent prefusion F vaccine administered during pregnancy are already approved for the protection of infants from RSV illness [22, 23, 24, 25, 26].

The advent of new interventions against RSV will inevitably increase the need for data on RSV burden to inform the required cost‐effectiveness analyses and to guide the optimal use of the products [27, 28, 29, 30]. Because the rates of RSV hospitalization are clearly highest during the first months of life and because the duration of protection afforded by an intervention may also be limited to a few months after birth, detailed data on illness burden especially during this period of the greatest vulnerability of infants will be important [31]. Several studies with different designs have assessed the rates of RSV hospitalization in infants <6 or <12 months of age [4, 5, 6, 7, 8, 9, 10, 11, 12], but few studies have reported the rates of hospitalization in monthly age groups of young infants [13, 14]. The purpose of this 10‐year analysis was to determine the average population‐based incidence rates of RSV hospitalization in children, with a special focus on monthly age groups of infants during the first year of life.

## 2 Methods

### 2.1 Study Design and Subjects

This retrospective study was performed at the Department of Pediatrics, Turku University Hospital, Finland, during the 10‐year period of September 1, 2008, through August 31, 2018.

The study population consisted of all children ≤15 years of age who were hospitalized with virologically confirmed RSV infection at Turku University Hospital on any day during the 10‐year period. For reliable estimation of population‐based rates of hospitalization, only children who lived within the catchment area of the hospital that served as the sole provider of acute care for children were included in the analyses. During the 10‐year study period, the average population of children ≤15 years of age living in the catchment area was 78,726 (Table S1).

### 2.2 Sources of Data

To find all children admitted with virologically confirmed RSV infection, we searched both the databases of the Department of Virology at the University of Turku and the central database of Turku University Hospital. Any duplicate admissions were removed by checking the unique person identifier numbers of the children. All data on clinical variables, management, and outcomes were collected by a structured review of the medical records. Detailed data on annual numbers of children in different age groups living in the catchment area of the hospital were retrieved from the official databases of Statistics Finland (Table S1).

### 2.3 Viral Diagnosis

During the study period, nasopharyngeal sampling for determination of the viral etiology of the illness was routine for all children hospitalized with respiratory symptoms. The virologic diagnosis of RSV was based on RT‐PCR and/or antigen detection assays. Identification of RSV by any method was considered to indicate an RSV‐associated hospitalization.

### 2.4 Definitions

The duration of hospitalization was recorded as the number of nights spent on the ward. In the rare cases when a child was admitted to a ward in the morning but discharged already in the evening of the same day, the duration of hospitalization was recorded as 1 day. Rehospitalization was defined as an unplanned readmission to the hospital for the same condition without the disappearance of symptoms in the meantime. For the purposes of this study, 18 children who had two distinct RSV infections during the study period were considered separate children, and they were analyzed in the age group that they belonged to at the time of admission. Children were considered to have an underlying condition if they had a chronic lung disease, congenital heart disease, immunosuppressive condition, neuromuscular disorder, genetic disorder, or intellectual disability that affected their ability to clear mucus secretions. Respiratory distress included any signs of breathing difficulty (e.g., intercostal or jugular retractions, tachypnea, wheezing, or nasal flaring) observed during the physical examination and documented in the medical records.

### 2.5 Statistical Methods

The incidence rates of RSV hospitalization in different age groups were calculated by dividing the numbers of RSV‐associated hospitalizations by the age‐specific populations, and they were expressed as average annual rates per 1000 children. Calculation of 95% confidence intervals (CIs) for incidence rates was based on the Poisson distribution. Comparison of proportions between groups was performed by the χ^2^ test, and Kruskal–Wallis test was used for comparing non‐normally distributed continuous data between several groups. Two‐sided *p* values <0.05 were considered to indicate statistical significance. All statistical analyses were performed with StatsDirect, Version 3.3.4 (StatsDirect, Wirral, UK).

## 3 Results

### 3.1 RSV Hospitalizations and Patient Demographics

**FIGURE 1:**
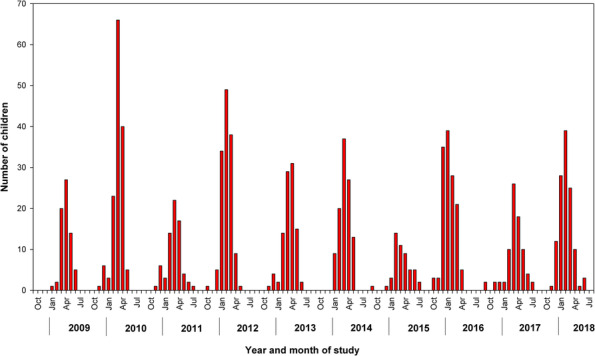
Numbers of children hospitalized with virologically confirmed respiratory syncytial virus infections during each month of the 10‐year study period.

A total of 1006 children were hospitalized with RSV during the 10‐year study period. Of these hospitalizations, 716 (71.2%) occurred between October through March, and 909 (90.4%) between October through April (Figure 1). Of all children with RSV‐associated hospitalization, 469 (46.6%) were <3 months of age, 634 (63.0%) were <6 months of age, and 747 (74.3%) were <1 year of age (Table 1). Boys accounted for 550 (54.7%) of all hospitalizations. A total of 96 (9.5%) children were born before 37 weeks of gestation, and 107 (10.6%) children had an underlying condition; the proportion of children with an underlying condition increased with age.

**TABLE 1:** Characteristics of 1006 children hospitalized with respiratory syncytial virus infection.

Characteristic	No. of children (%)
Age	
0–<3 months	469 (46.6)
3–<6 months	165 (16.4)
6–<12 months	113 (11.2)
1–<2 years	120 (11.9)
2–<5 years	112 (11.1)
5–15 years	27 (2.7)
Sex	
Male	550 (54.7)
Female	456 (45.3)
Gestational age (week)	
≥37	910 (90.5)
34–<37	52 (5.2)
32–<34	16 (1.6)
28–<32	14 (1.4)
<28	14 (1.4)
Underlying condition	
0–<3 months	9/469 (1.9)
3–<6 months	8/165 (4.8)
6–<12 months	15/113 (13.3)
1–<2 years	20/120 (16.7)
2–<5 years	40/112 (35.7)
5–15 years	15/27 (55.6)

### 3.2 Incidence Rates of RSV‐Associated Hospitalizations

In monthly age groups during the first year of life, the average annual population‐based rate of RSV hospitalization was highest among infants 1 month of age (52.0 per 1000 children; 95% CI, 45.2–59.7), followed by infants <1 month of age (34.8 per 1000; 95% CI, 29.2–41.1) and those 2 months of age (32.2 per 1000; 95% CI, 26.9–38.4; Table 2). In cumulative age groups, the rate of hospitalization was 39.7 per 1000 (95% CI, 36.2–43.4) among all infants <3 months of age, 26.8 per 1000 (95% CI, 24.8–29.0) in infants <6 months of age and 15.8 per 1000 (95% CI, 14.7–17.0) in those <12 months of age.

**TABLE 2:** Average annual rates of RSV‐associated hospitalizations per 1000 children among different age groups.

Age group	No. of children	Rate per 1000	95% CI
<1 (month)	137	34.8	29.2–41.1
1	205	52.0	45.2–59.7
2	127	32.2	26.9–38.4
3	63	16.0	12.3–20.5
4	67	17.0	13.2–21.6
5	35	8.9	6.2–12.4
6	37	9.4	6.6–12.9
7	16	4.1	2.3–6.6
8	19	4.8	2.9–7.5
9	13	3.3	1.8–5.6
10	11	2.8	1.4–5.0
11	17	4.3	2.5–6.9
1 (year)	120	2.5	2.1–3.0
2	69	1.4	1.1–1.8
3	28	0.6	0.4–0.8
4	15	0.3	0.2–0.5
5–9	16	0.07	0.04–0.11
10–15	11	0.04	0.02–0.07
0–<3 months	469	39.7	36.2–43.4
0–<6 months	634	26.8	24.8–29.0
0–<9 months	706	19.9	18.5–21.4
0–<12 months	747	15.8	14.7–17.0
0–<2 years	867	9.1	8.5–9.7
0–< 5 years	979	4.1	3.8–4.3
0–15 years	1006	1.3	1.2–1.4

### 3.3 Clinical Features and Complications

A total of 808 (80.3%) children had signs of respiratory distress, and the proportion of such children peaked at 2–5 months of age (Table 3). Pneumonia was diagnosed in 62 (8.3%) children <1 year of age and in 106 (40.9%) children 1–15 years of age (*p* < 0.0001). The diagnosis of acute otitis media was made in 351 (47.0%) children <1 year of age, with the proportion decreasing with increasing age. Overall, 589 (58.5%) children received antibiotic treatment during their hospitalization.

**TABLE 3:** Clinical features and management of 1006 children hospitalized with respiratory syncytial virus infection.

Variable	Age group								
	<1 month (*n* = 137)	1 month (*n* = 205)	2 months (*n* = 127)	3–5 months (*n* = 165)	6–11 months (*n* = 113)	1 year (*n* = 120)	2–4 years (*n* = 112)	5–15 years (*n* = 27)	All (*n* = 1006)
Respiratory distress	111 (81.0)	177 (86.3)	118 (92.9)	155 (93.9)	90 (79.6)	84 (70.0)	66 (58.9)	7 (25.9)	808 (80.3)
Pneumonia	16 (11.7)	15 (7.3)	7 (5.5)	9 (5.5)	15 (13.3)	43 (35.8)	51 (45.5)	12 (44.4)	168 (16.7)
Acute otitis media	56 (40.9)	92 (44.9)	57 (44.9)	83 (50.3)	63 (55.8)	47 (39.2)	36 (32.1)	2 (7.4)	436 (43.3)
Antibiotic treatment	73 (53.3)	114 (55.6)	65 (51.2)	93 (56.4)	71 (62.8)	77 (64.2)	76 (67.9)	20 (74.1)	589 (58.5)
Duration of hospitalization, mean (SD), d	3.8 (2.6)	3.4 (3.4)	2.7 (2.2)	2.3 (1.8)	3.3 (7.9)	2.3 (2.5)	2.5 (3.3)	5.7 (8.6)	3.0 (4.0)
Duration of hospitalization, median (IQR), d	3 (2–5)	3 (2–4)	2 (1–3)	2 (1–3)	1 (1–3)	2 (1–3)	2 (1–3)	3 (1–4)	2 (1–4)
Rehospitalization	7 (5.1)	14 (6.8)	5 (3.9)	8 (4.8)	3 (2.7)	5 (4.2)	12 (10.7)	1 (3.7)	55 (5.5)
Intensive care treatment	34 (24.8)	26 (12.7)	13 (10.2)	10 (6.1)	7 (6.2)	15 (12.5)	8 (7.1)	4 (14.8)	117 (11.6)
Mechanical ventilation	3 (2.2)	2 (1.0)	2 (1.6)	1 (0.6)	1 (0.9)	3 (2.5)	0	1 (3.7)	13 (1.3)
Death	0	0	0	0	0	2 (1.7)	1 (0.9)	1 (3.7)	4 (0.4)

*Note:* Data are *n* (%) unless otherwise indicated.

Abbreviations: IQR, interquartile range; SD, standard deviation.

### 3.4 Duration of Hospitalization

Among all children, the median duration of hospitalization was 2.0 days (interquartile range [IQR], 1.0–4.0 days), while the mean duration was 3.0 days (SD, 4.0 days; Table 3). The median duration of hospitalization in infants <3 months of age was 3.0 days (IQR, 1.0–4.0 days), which was significantly longer than the corresponding durations in age groups 3–5 months (2.0 days; IQR, 1.0–3.0 days), 6–11 months (1.0 days; IQR, 1.0–3.0 days), or 1–15 years (2.0 days; IQR, 1.0–3.0 days; *p* < 0.0001 for comparisons between infants <3 months and any of the older age groups).

### 3.5 Treatment at Pediatric Intensive Care Unit

A total of 117 (11.6%) children were treated at the pediatric intensive care unit (PICU) (Table 3). The frequency of intensive care treatment was highest (24.8%) among infants <1 month of age. In cumulative age groups, the proportions of children treated at the PICU were 15.6% in infants <3 months of age, 13.1% in infants <6 months of age, and 12.0% among those <12 months of age. Treatment at the PICU was more frequent among children born prematurely (24 of 96, 25.0%) than in children born full‐term (93 of 910, 10.2%; *p* < 0.0001). No significant difference was observed in the frequency of PICU treatment between children with underlying conditions (14 of 107, 13.1%) and those without (103 of 899, 11.5%; *p* = 0.62). Thirteen (1.3%) children were mechanically ventilated. Four (0.4%) children died; all of them were ≥1 year of age and had a severe underlying neuromuscular disorder.

## 4 Discussion

Our 10‐year study provides detailed and comprehensive data on population‐based incidence rates of RSV hospitalization in different age groups of children. The most important finding of our study is that especially during the first 3 months of life, the rates of RSV hospitalization appeared to be substantially higher than those reported previously. Numerous studies with different designs have determined RSV hospitalization rates in various age groups of children, usually among children <6 or <12 months of age [4, 5, 6, 7, 8, 9, 10, 11, 12], but only two previous studies have reported population‐based rates of hospitalization in monthly age groups of infants [13, 14]. The study by Hall et al. [13] covered five respiratory seasons from October through March, whereas the study by Rha et al. [14] included one winter season. The RSV hospitalization rates in monthly age groups of infants in those two US studies and in the present study are presented in Figure S1. All these studies share the same general pattern, with the highest age‐specific rate observed in infants 1 month of age. However, the absolute rates of RSV hospitalization in the present study were approximately twofold higher than in the previous studies among infants up to 3 months of age.

Several differences in the design and execution of the studies may explain the observed differences. In the previous prospective studies [13, 14], participation required an informed consent from the parents, and eventually only 64%–85% of eligible children could be enrolled in those studies. Because in our study RSV testing was routine practice in all children hospitalized with respiratory infections, we could identify all RSV‐positive children without the need to obtain a separate informed consent, and sampling was not limited to certain hours of the day or days of the week. Although it is well established that the circulation of RSV is most prominent during the winter months in temperate regions, it is noteworthy that in our study, approximately 30% of all RSV hospitalizations occurred outside of the period from October through March and 10% even outside the period from October through April.

Obviously, it is possible that the differences observed between the studies would reflect true differences in RSV hospitalization rates of infants between the countries. However, at least two findings would argue against a notion that the differences could be explained by varying thresholds for hospitalization. First, in a recent large prospective birth cohort study in Europe, the rate of RSV hospitalization among infants in five European countries was lowest in Finland [5]. Second, the RSV hospitalization rates among infants <6 months of age in our study were similar to the average rates observed in a recent meta‐analysis of studies of RSV hospitalizations in the United States [7]. In that meta‐analysis, the rates reported in active surveillance studies were approximately half those from studies based on ICD codes. The high specificity of RSV‐specific ICD codes for true RSV illnesses has been demonstrated in some previous studies [32, 33]. Therefore, although active surveillance studies in well‐defined populations could theoretically provide high‐quality evidence on RSV admissions, it is likely that challenges in, for example, recruitment, obtaining informed consent, and sampling for viruses at any time may lead to substantial underestimation of RSV hospitalization rates in infants [7].

Accurate data on population‐based incidences of RSV hospitalization especially among infants during their first months of life are of crucial importance to inform decision‐making at this time when prophylactic interventions against RSV are becoming available [27, 28, 29, 30, 31]. As the effectiveness of RSV prevention by either maternal vaccination or the administration of monoclonal antibodies with extended half‐life to infants is greatest during a couple of months after the birth, reliable data on the burden of illness during those months when also the incidence of RSV hospitalization is highest would be most important for cost‐effectiveness evaluations of various interventions.

Besides the rates of RSV hospitalization, the duration of hospitalization and the frequency of intensive care treatment are important factors in assessing the cost‐effectiveness of RSV interventions. In our study, the median length of stay was 2 days, but studies in other countries have reported substantially longer durations of up to 5 days [34, 35, 36, 37]. It is unlikely that the differences in the average length of stay between different countries would be indicative of true differences in the severity of RSV illnesses, but most probably they reflect local ways and traditions of managing hospitalized children. Nevertheless, because the duration of hospitalization is directly related to the overall cost of hospitalization, knowledge of the local durations would be important for policymakers in each country. The same applies to treatment at the intensive care unit, which is usually the most expensive part of hospitalization.

The main strengths of our study include the long observation period that balanced any year‐to‐year variation in the epidemiology of RSV, routine testing for RSV in all children hospitalized with respiratory infections, and the well‐defined catchment population. Owing to routine sampling for viruses, our analyses were not restricted to the conventional respiratory seasons, but we were able to include all RSV‐positive children admitted at any time or day during the entire 10‐year period. Our study has also some limitations. Although obtaining viral specimens from hospitalized children was a routine procedure at our hospital during the study period, it is possible that some children with RSV illness had not been subjected to viral sampling. Moreover, some of the obtained tests may have remained false negative. However, for these reasons, our population‐based rates should be considered as conservative estimates, and the true rates of RSV hospitalization might be even slightly higher than those observed in the present analysis. Because the management of children hospitalized with RSV varies between different countries, our results are generalizable only to Finland and countries that have similar practices of hospitalization. Our study was performed at a single tertiary care hospital, which restricted the size of the study population; inclusion of other hospitals with routine viral diagnostic practices might have increased the accuracy of the estimates of RSV hospitalization rates in different age groups.

In conclusion, our results demonstrate that the population‐based incidence rates of RSV hospitalization among infants during the first months of life may be substantially higher than those reported in earlier studies. The present data could allow for a more precise estimation of the cost‐effectiveness of various interventions aiming at reducing the overall burden of RSV in infants and children. However, considering the relative scarcity of population‐based data on virologically confirmed RSV hospitalizations among young infants, it is clear that further studies in different countries and areas are warranted.

## Author Contributions

**Erika Uusitupa:** Conceptualization; Data curation; Formal analysis; Writing – original draft; Writing – review and editing. **Matti Waris:** Formal analysis; Investigation; Methodology; Writing – review and editing. **Tytti Vuorinen:** Formal analysis; Investigation; Methodology; Writing – review and editing. **Terho Heikkinen:** Conceptualization; Data curation; Formal analysis; Investigation; Methodology; Project administration; Supervision; Writing – original draft; Writing – review and editing.

## Conflicts of Interest

TH has received honoraria for lectures or participation in advisory boards or data monitoring committees from Janssen, Sanofi, Enanta, MSD, Novavax, Moderna, and Pfizer. The other authors declare no conflicts of interest.

### Peer Review

The peer review history for this article is available at https://www.webofscience.com/api/gateway/wos/peer‐review/10.1111/irv.13268.

## Supporting information


**Table S1.** Numbers of children in different age groups living in the catchment area of Turku University Hospital during the 10‐year study period.

**Figure S1.** Respiratory syncytial virus‐associated hospitalizations in monthly age groups of infants. Data for Hall et al. were derived from *Pediatrics 2013;132:e341‐8*, and data for Rha et al. were derived from *Pediatrics 2020;146:e20193611*. Uusitupa et al. denotes the present study.

## Data Availability

The data that support the findings of this study are available on request from the corresponding author. The data are not publicly available due to privacy or ethical restrictions.
