# Supplementary material for: Respiratory Syncytial Virus‐Associated Hospitalizations in Children: A 10‐Year Population‐Based Analysis in Finland, 2008–2018
Source: Influenza Other Respir Viruses. 2024 Mar 13;18(3):e13268. doi: 10.1111/irv.13268 (PMC10934253; doi:10.1111/irv.13268)
Supplement: Supplementary file 1 — Table S1. Numbers of children in different age groups living in the catchment area of Turku University Hospital during the 10‐year study period. [file IRV-18-e13268-s002.docx]

**Supplementary Table 1.**

Numbers of children in different age groups living in the catchment area of Turku University Hospital during the 10-year study period.

| **Age (years)** | **Season** | | | | | | | | | | **10-year average population** |
| --- | --- | --- | --- | --- | --- | --- | --- | --- | --- | --- | --- |
|  | **2008-09** | **2009-10** | **2010-11** | **2011-12** | **2012-13** | **2013-14** | **2014-15** | **2015-16** | **2016-17** | **2017-18** |  |
| **0*** | 4880 | 4911 | 5112 | 4918 | 4811 | 4756 | 4776 | 4615 | 4311 | 4185 | 4727,5 |
| **1** | 4799 | 4868 | 4940 | 5127 | 4959 | 4831 | 4759 | 4800 | 4631 | 4347 | 4806,1 |
| **2** | 4791 | 4824 | 4884 | 4947 | 5130 | 4972 | 4837 | 4779 | 4797 | 4695 | 4865,6 |
| **3** | 4686 | 4800 | 4835 | 4902 | 4975 | 5140 | 4994 | 4838 | 4791 | 4827 | 4878,8 |
| **4** | 4875 | 4704 | 4803 | 4842 | 4932 | 4969 | 5130 | 4997 | 4855 | 4816 | 4892,3 |
| **5** | 4687 | 4881 | 4741 | 4813 | 4860 | 4966 | 4989 | 5132 | 5003 | 4884 | 4895,6 |
| **6** | 4662 | 4704 | 4900 | 4765 | 4828 | 4888 | 4981 | 5025 | 5152 | 5031 | 4893,6 |
| **7** | 4742 | 4691 | 4726 | 4909 | 4763 | 4850 | 4910 | 4983 | 5026 | 5203 | 4880,3 |
| **8** | 4928 | 4747 | 4698 | 4753 | 4934 | 4779 | 4860 | 4918 | 5003 | 5052 | 4867,2 |
| **9** | 5027 | 4949 | 4764 | 4717 | 4785 | 4965 | 4795 | 4893 | 4913 | 5026 | 4883,4 |
| **10** | 4984 | 5044 | 4966 | 4783 | 4726 | 4805 | 4989 | 4816 | 4883 | 4916 | 4891,2 |
| **11** | 5155 | 4980 | 5062 | 4966 | 4822 | 4761 | 4813 | 5006 | 4835 | 4925 | 4932,5 |
| **12** | 5248 | 5171 | 5008 | 5076 | 4989 | 4844 | 4761 | 4821 | 5011 | 4859 | 4978,8 |
| **13** | 5355 | 5263 | 5173 | 5004 | 5082 | 5020 | 4863 | 4784 | 4834 | 5024 | 5040,2 |
| **14** | 5542 | 5364 | 5281 | 5183 | 5022 | 5116 | 5046 | 4883 | 4793 | 4856 | 5108,6 |
| **15** | 5469 | 5552 | 5379 | 5293 | 5196 | 5047 | 5137 | 5066 | 4899 | 4805 | 5184,3 |

* For calculation of the annual rates of RSV-associated hospitalizations in monthly age groups of infants during the first year of life, the average population was divided by 12.
