# Supplementary material for: Respiratory Syncytial Virus‐Associated Hospitalizations in Children: A 10‐Year Population‐Based Analysis in Finland, 2008–2018
Source: Influenza Other Respir Viruses. 2024 Mar 13;18(3):e13268. doi: 10.1111/irv.13268 (PMC10934253; doi:10.1111/irv.13268)

Supplemental Figure 1. Respiratory syncytial virus-associated hospitalizations in monthly age groups of infants. Data for Hall et al. were derived from *Pediatrics 2013;132:e341-8*, and data for Rha et al. were derived from *Pediatrics 2020;146:e20193611*. Uusitupa et al. denotes the present study.

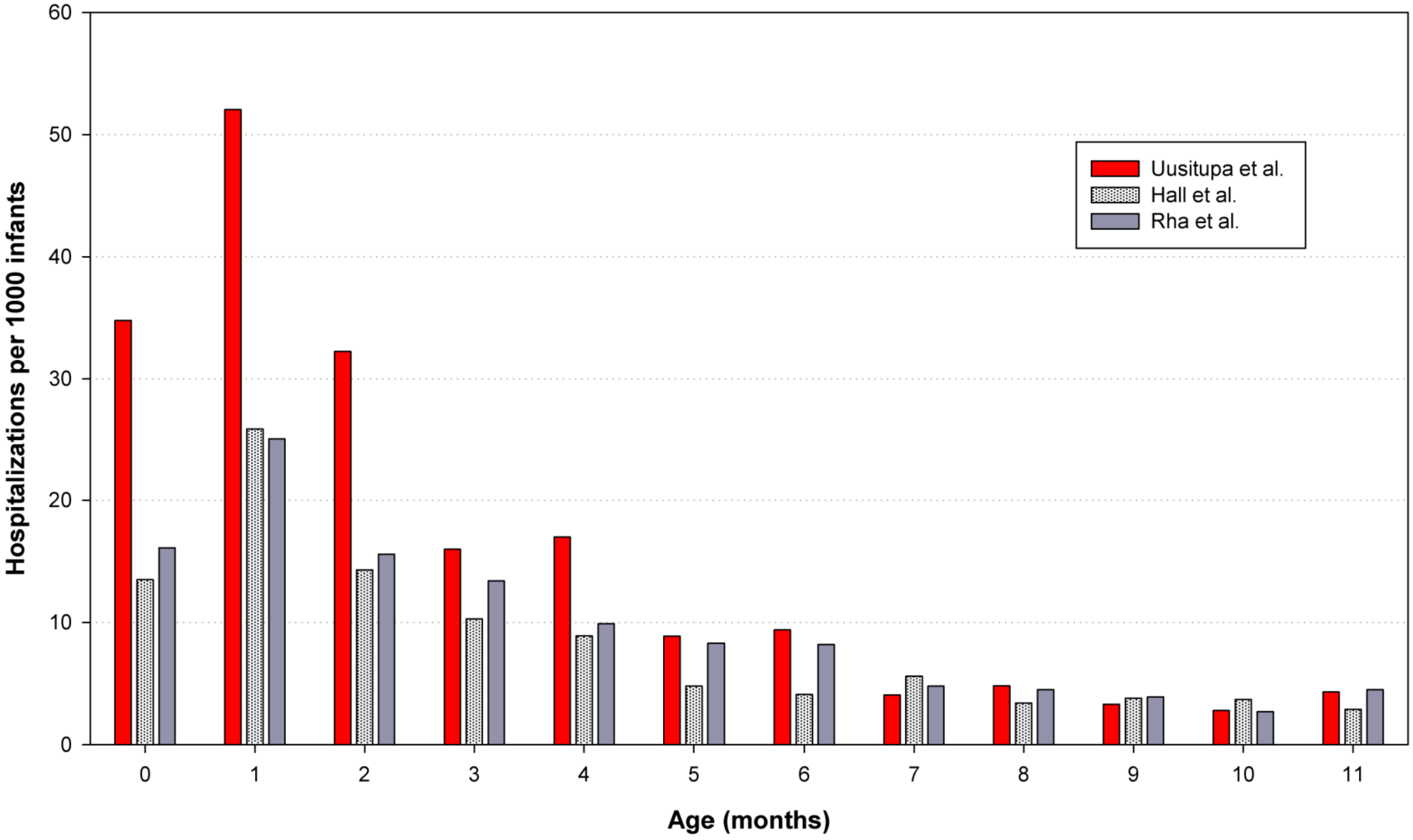

Supplement: Supplementary file 2 — Figure S1. Respiratory syncytial virus‐associated hospitalizations in monthly age groups of infants. Data for Hall et al. were derived from Pediatrics 2013;132:e341‐8, and data for Rha et al. were derived from Pediatrics 2020;146:e20193611. Uusitupa et al. denotes the present study. [file IRV-18-e13268-s001.pdf]
